# Purification and Characterization of Pathogenesis Related Class 10 Panallergens

**DOI:** 10.3390/foods8120609

**Published:** 2019-11-22

**Authors:** Jane K. McBride, Hsiaopo Cheng, Soheila J. Maleki, Barry K. Hurlburt

**Affiliations:** US Department of Agriculture, Agricultural Research Service, Southern Regional Research Center, New Orleans, LA 70124, USA; jane.mcbride@usda.gov (J.K.M.); hsiaopo.cheng@usda.gov (H.C.); soheila.maleki@usda.gov (S.J.M.)

**Keywords:** panallergen, pollen, peanut, hazelnut, protein purification, structure, ligand binding, IgE binding, flavonoids

## Abstract

Oral allergy syndrome (OAS) describes an allergic reaction where an individual sensitized by pollen allergens develops symptoms after eating certain foods. OAS is caused by cross-reactivity among a class of proteins ubiquitous in plants called pathogenesis related class 10 (PR-10) proteins. The best characterized PR-10 protein is Bet v 1 from birch pollen and its putative function is binding hydrophobic ligands. We cloned a subset of seven recombinant PR-10 proteins from pollens, peanuts, and hazelnuts and developed a standard purification method for them. Immunoglobulin E (IgE) binding of purified PR-10 proteins was analyzed by ImmunoCAP ISAC microarray and enzyme-linked immunosorbent assays (ELISAs) with sera from allergic patients. We investigated the binding activities of PR10s by testing 16 different ligands with each protein and compared their secondary structures using circular dichroism (CD). The PR-10s in this study had very similar CD spectra, but bound IgE with very different affinities. All seven proteins showed a similar pattern of binding to the polyphenol ligands (resveratrol, flavonoids, and isoflavones) and variable binding to other potential ligands (fatty acids, sterols, and plant hormones). We suggest our protocol has the potential to be a near-universal method for PR-10 purification that will facilitate further research into this important class of panallergens.

## 1. Introduction

Oral allergy syndrome (OAS) is a condition wherein an individual who has been sensitized to tree or weed pollen develops an Immunoglobulin E (IgE)-mediated reaction upon eating certain fresh fruits or other foods. The allergic reaction elicited by the foods is generally confined to the oral mucosa, and symptoms include itching, swelling and burning of lips, tongue and throat, although rarely a more severe reaction may result, such as anaphylaxis [1]. OAS is mainly caused by the immunological cross-reactivity between pollen allergens belonging to the ubiquitous pathogenesis-related protein-10 (PR-10) family and their homologs in plant foods, notably members of the Rosacae family (apples, pears, peaches, and cherries), celery, legumes, and tree nuts [2,3,4,5,6,7]. The best-characterized member of this protein family is Bet v 1 from *Betula verrucosa* (white birch) pollen, which is a major cause of seasonal allergies in Central Europe and North America [8].

PR-10 proteins are small in size (16–17 kDa) and although they often have quite divergent primary sequences [9], all share a highly-conserved, unusual structure that consists of a seven-stranded antiparallel beta-sheet and a long C-terminal alpha-helix that forms a large forked hydrophobic cavity [10]. As most plant PR-10 proteins are labile in structure and readily susceptible to inactivation of their IgE binding ability through heat, processing, or pepsin digestion [4,11], they generally only elicit responses from raw foods or environmental inhalants. Their placement within the large PR protein family of plant defensive genes is due to their accumulation around pathogen invasion sites and physical wounds as well as induction by other stress factors like drought, cold, and salinity, however, their mechanism of response is not well understood [12]. They have a glycine-rich motif that resembles the P-loop found in nucleotide-binding proteins [10], and several PR-10 proteins have been shown to possess RNase activity [13,14], however, it does not seem to be a universal characteristic. There is widespread evidence of general anti-pathogen (bacterial, viral, fungal, and parasitic) activity but the mechanisms are largely unknown [15], except for one report of a PR-10 acting as a protease inhibitor [16].

The best-characterized function of PR-10 proteins is binding small hydrophobic ligands (such as fatty acids, flavonoids, and cytokinins) within the large internal cavity. Biological ligands of PR-10 proteins have been identified through in vitro screens utilizing a fluorescent ligand, 8-anilino-1-napthalenesulfonic acid (ANS), which is displaced from the cavity upon the addition of a binding partner [17,18], as well as co-crystallization or solution structures of PR-10s bound to one or more ligands [17,18,19,20,21,22,23,24]. As flavonoids and cytokinins are important cell-cycle regulators and chemical messengers [25,26], their binding, transport, and sequestration within the cell could be the primary mechanism by which PR-10 proteins mediate the stress response [19,24]. The similarity of the structures of PR-10 proteins to the StAR-related lipid transfer (START) domain of the human cholesterol transporter, MLN64 (metastatic lymph node 64 protein), suggests the evolutionary importance and universality of this protein fold [27].

This study aims to compare the requirements for purification, secondary structures, and binding to human IgE and potential biological ligands of a subset of recombinant PR-10 proteins. Recombinant Bet v 1.01 (referred to hence as rBet v 1) is included as it is the best-studied member of the family and the most commonly recognized amongst PR-10-allergic individuals [28,29]. Also included are two isoforms each of PR-10s from hazel (rCor a 1.02 from hazel pollen and rCor a 1.04 from hazelnut), white oak (rQue a 1.02 and rQue a 1.03), and peanut (rAra h 8.01 and rAra h 8.02). The hazel pollen PR-10 from European hazelnut (*Corylus avellana*), Cor a 1.01, is the second-most commonly recognized PR-10 protein in both birch-endemic and birch-free regions, like the Mediterranean [28,29]. In other birch-free regions, the PR-10 proteins from white oak (*Quercus alba*) and other oak species are important sensitizing agents of OAS [30]. OAS elicited by the Bet v 1 homologs of peanut (*Arachis hypogaea*), the Ara h 8 isoforms, is often not confined to the oral cavity, and can manifest as serious symptoms like vomiting, hives, and difficult breathing. Moreover, the peanut PR-10 proteins seem to be less heat-labile than other Bet v 1 homologs, as natural Ara h 8 from roasted peanut has been shown to have enhanced IgE-binding ability and stability [31], though some studies show a reduction in allergenicity [4]. As most peanut products consumed by humans are heat-treated, this suggests that Ara h 8 has a more stable structure and could potentially be a more potent allergen than more widely-recognized PR-10s from fruits and vegetables which are consumed raw.

**Figure 1:**
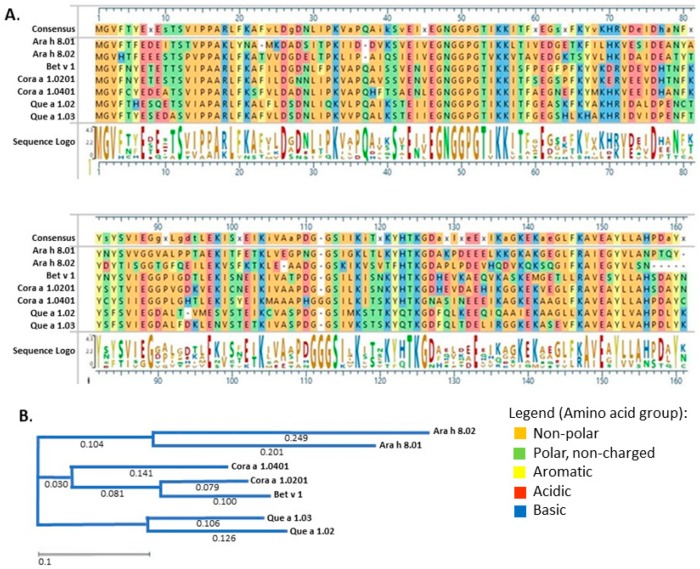
(**A**) Alignment and (**B**) phylogenetic tree of selected pathogenesis-related protein-10 (PR-10) protein amino acid sequences. The amino acid sequences of the seven PR-10 proteins presented in this study were entered into MegAlign Pro (DNASTAR Lasergene) and aligned using the Clustal Omega algorithm.

An alignment of amino acid sequences of these seven proteins reveals that the Ara h 8 isoforms are the most divergent from the rest and each other, the oak pollen allergens are most closely related to each other, and the hazel proteins are the most similar to Bet v 1 (Figure 1). The most obvious difference in the seven amino acid sequences is the deletion of the last five amino acids in Ara h 8.02. Cor a 1.04 has an extra glycine inserted in the conserved GG found at position 111–113. There are variable regions of four amino acids also found at positions 62–65 and 128–131, and both occur just after conserved glutamic acid residues.

All of the PR-10 proteins presented here were expressed and purified in an almost identical fashion. They all showed similar secondary structures and bound many of the tested ligands, though with varying affinities. All of the proteins were able to bind human IgE as measured by a commercial solid-phase multiplex binding assay (ImmunoCAP ISAC array) and by individually testing proteins by enzyme-linked immunosorbent assay (ELISA) with sera from allergic patients. As these proteins appear to retain their structure, immunological reactivity and biological function, we suggest our purification protocol can serve as a general guide to facilitate purification of virtually any recombinant PR-10 protein with only minor adjustments necessary. These proteins can be used in a variety of studies into structure and function of this important class of panallergens.

## 2. Materials and Methods

### 2.1. Patient Sera

Sera from allergic individuals were collected after informed consent at Tulane University (New Orleans, LA, USA) in accordance with the rules and regulations of the institutional review board (Food Allergy Sera Bank (09-00231)). Information about patients and their specific IgE (sIgE) in kU_A_/L to relevant PR-10 proteins as determined by ImmunoCAP ISAC microarray (Thermo Fisher Scientific, Phadia AB, Uppsala, Sweden) is shown in Table 1.

### 2.2. Cloning

Amino acid sequences of the PR-10 proteins presented in this study were retrieved from the Allergome database, with the most representative isoforms of each species being chosen for cloning, and were reverse transcribed using SeqBuilder Pro (DNAStar Lasergene, Madison, WI, USA). Synthetic geneblocks encoding each PR-10 transcript were designed with optimal codon usage for *Escherichia coli* (Integrated DNA Technologies, Skokie, IL, USA). Polymerase chain reaction (PCR) amplification was performed with primers incorporating 5’ Nde I and 3’ Bam HI restriction enzyme sites using the high-fidelity Phusion polymerase (New England Biolabs, Ipswich, MA, USA). Following PCR amplification, products and the expression vector pET9a (Promega, Madison, WI, USA) were digested with Nde I and Bam HI High Fidelity restriction endonucleases and ligated with T4 ligase (New England Biolabs, Ipswich, MA, USA). Transformed DH5α colonies were selected on Luria-Bertani (LB) plates containing kanamycin. Miniprep DNA was obtained from overnight cultures with Qiagen Miniprep kit (Qiagen, Germantown, MD, USA). All constructs were confirmed by DNA sequencing.

### 2.3. Expression and Purification of Recombinant PR-10 Proteins

The protocol for each protein was modified from the protocol developed for rAra h 8.01 [18]. *E. coli* expression strain BL21 (DE3) was transformed with pET9a-PR-10 clones. We used 10 mL of overnight culture to inoculate one liter of LB-kanamycin in 2 L baffled flasks. Culture flasks were incubated at 37 °C with shaking at 150 rpm until the optical density (O.D.) reached 0.5. Protein expression was induced by adding 0.5 mM isopropyl β-D-1-thiogalactopyranoside (IPTG), and cultures were incubated either at 37 °C for three hours or at 16 °C overnight depending on the solubility of the expressed protein. Cultures were harvested by centrifugation at 3000× *g*. Cell pellets were resuspended in Q Column Buffer (QCB) -50 buffer (20 mM Tris-Cl pH 8.4, 50 mM NaCl, 1 mM EDTA) plus 1 mM protease inhibitor, phenylmethylsulfonyl fluoride (PMSF), and 1 mM dithiothreitol (DTT) on ice. Lysis was achieved by adding lysozyme to a final concentration of 150 ng/mL, and the lysate was cleared by sonication and centrifugation at 10,000× *g* for 20 min. The cleared lysate was applied to the High-Q strong anion exchange resin (BioRad, Hercules, CA, USA) and the flow-through and wash was collected and brought to just below point of precipitation of the recombinant protein with ammonium sulfate (see Table 2). After clearing the precipitated proteins by centrifugation, the supernatant was applied to n-butyl FF column (GE Healthcare Life Sciences, Pittsburg, PA, USA) on an AKTA Purifier FPLC (fast protein liquid chromatography) and the PR-10 protein was eluted by a reverse salt gradient. Fractions were analyzed by sodium dodecyl sulfate-polyacrylamide gel electrophoresis (SDS-PAGE) and those containing the recombinant PR-10 were combined, concentrated and dialyzed into QCB-50 buffer. For circular dichroism (CD) analysis, concentrated PR-10s were loaded onto the Superdex 200 size exclusion column (GE Healthcare Life Sciences, Chicago, IL, USA) and eluted into ultrapure water. The isoelectric points (pI) and molecular weights (MW) of each protein (Table 2) were calculated using Protean 14 (DNAStar Lasergene, Madison, WI, USA).

### 2.4. Liquid Chromatography-Mass Spectrometry (LC-MSMS)

In-gel digestion with trypsin was carried out using the In-Gel Tryptic Digestion Kit (Thermo-Fisher Scientific, Waltham, MA, USA) according to the manufacturer’s instructions. Briefly, bands were destained in 200 mM ammonium bicarbonate, washed with 50:50 acetonitrile:water, reduced with 10 mM TCEP in 50 mM ammonium bicarbonate (pH 8.6) buffer for 10 minutes at 60 °C, and alkylated in 55 mM iodocetamide in 100 mM ammonium bicarbonate for one hour. Trypsin in ammonium bicarbonate at 20 ng/µL was added to cover gel pieces and incubated overnight at 30 °C. The digests were analyzed via LC-MSMS, using an Agilent 1200 LC system, an Agilent Chip-cube interface and an Agilent 6520 Q-TOF tandem mass spectrometer (Agilent Technologies, Santa Clara, CA, USA). Data files were transferred to an Agilent workstation equipped with Spectrum Mill software (Agilent Technologies, Santa Clara, CA, USA). The raw MS/MS data files were extracted, sequenced, and searched against the seven amino acid sequences shown in Figure 1. The LC-MSMS data is shown in Figure S1.

### 2.5. IgE ELISA

Immulon 4 HBX polystyrene microtiter plates (Thermo Fisher, Waltham, MA, USA) were coated with 500 ng/well of purified protein in 0.1 M sodium carbonate buffer and incubated for 1 hour at 37 °C. Wells were washed three times with phosphate-buffered saline solution (PBS) containing 0.05% Tween-20 (PBST) and then blocked with 250 μL/well of PBS containing 3% non-fat milk for 2 h at 37 °C. The plate was washed as above and then patient sera diluted into an equal volume of PBS was added to each well and incubated for 1 h at 37 °C. After washing, 50 μL/well of a horseradish peroxidase (HRP)-conjugated mouse anti-human IgE (1:1000 dilution in PBS) (Sigma-Aldrich, St. Louis, MO, USA) was added and incubated for 1 h at 37 °C. After the plate was washed three times with PBST and once with PBS, the peroxidase reaction was developed with 100 μL of peroxidase substrate (SureBlue TM, KPL, Gaithersburg, MD, USA). After 30 min, the reaction was stopped with 100 μL of 0.03 N HCl, and the optical density (O.D.) was measured at 450 nm. The average O.D. reading from wells that were incubated with sera from a non-allergic individual was subtracted from each of the triplicate wells using allergic patients’ sera for each of the three proteins.

### 2.6. ANS Displacement Assay

All recombinant PR-10 proteins were tested for ligand binding by displacement of ANS as described by Mogensen et al. [17]. ANS was dissolved in 1 mL dimethylsulfoxide (DMSO) and diluted to 100 mL with ultrapure water, and its concentration was determined spectrophotometrically using an extinction co-efficient of 4990 M^−1^ cm^−1^ at 350 nm. Fluorescence experiments were performed on a Tecan ULTRA Evolution plate reader in black 96-well polypropylene plates (Thermo Scientific, Waltham, MA, USA). Fluorescence was measured by excitation at 360 nm and recording emission at 465 nm using an optimal gain and 40 µs integration time. Baseline fluorescence was defined as 40 uM ANS in the presence of equimolar purified PR-10 protein, and for each experiment the signal from four wells was averaged. For ligand binding experiments, each potential ligand was dissolved in an appropriate solvent (water, ethanol, or DMSO) to a concentration of 10 mM and added to the ANS-PR-10 reaction (40 uM ANS with 40 uM protein) step-wise in increments (from 20–300 uM competitor) and the fluorescence signal was measured after each addition. Signal strength was averaged over four wells. Relative strength of binding was analyzed by the method published previously for rAra h 8.01, where the percent change was calculated by dividing the average signal by the baseline fluorescence measured on the same plate [18].

### 2.7. t-Resveratrol Binding

Fluorescence was performed in a Shimadzu RF-5301 PC spectrophotometer and measured by excitation at 330 nm and recording emission from 335–600 nm. Baseline fluorescence of 40 uM *t*-resveratrol in 50 mM phosphate buffer, pH 7.4 was recorded, and then after the addition of purified PR-10 protein. Data was exported and plotted as signal intensity against wavelength in Origin 6.0 (OriginLab, Northampton, MA, USA)**.**

### 2.8. Far UV Circular Dichroism

Far UV (185–250 nm) circular dichroism spectra measurements were obtained in a Jasco Model J-710 spectropolarimeter (JASCO, Easton, MD, USA) in a cuvette with a path length of 0.1 cm. Purified PR-10 proteins eluted from a size exclusion column into ultra-pure water were analyzed, and the CD spectrum of ultra-pure water obtained as the background was subtracted from resulting spectra. All proteins were diluted to approximately 1 µM, and their spectra were measured three times at 20 °C, and the results were accumulated. Data was smoothed using a Savitzky–Golay filter.

## 3. Results

### 3.1. Recombinant Protein Expression and Purification

All of the recombinant PR-10 proteins presented here were highly expressed and soluble, but they are not universally able to be expressed in *E. coli*, as attempts to express two other isoforms from hazel were unsuccessful. Still the recombinant proteins in this study are similar enough that the same basic purification protocol was employed for all seven, with only the ammonium sulfate concentration varying slightly. A fortunate discovery was made which greatly aided the purification process for all PR-10s in this study: These proteins do not bind to the strong cation exchanger, High Q, unlike the majority of *E. coli* proteins. The initial step of passing the crude lysate through the Q resin and collecting the flow-through generally resulted in protein that was 50–70% pure.

**Figure 2:**
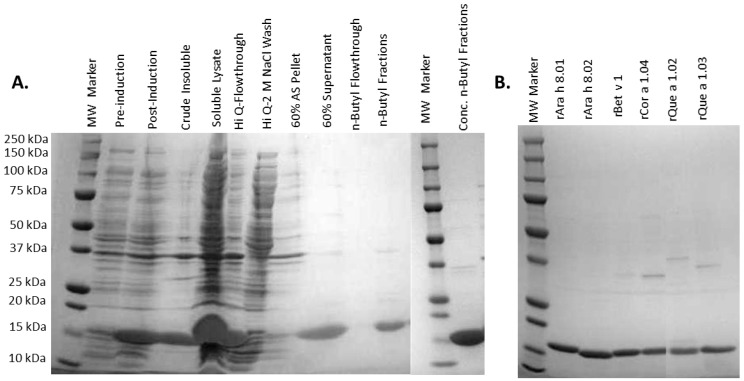
(**A**) The sodium dodecyl sulfate-polyacrylamide gel electrophoresis (SDS-PAGE) gel showing expression and purification steps for PR-10 protein rCor a 1.02. (**B**) The SDS-PAGE gel showing the pooled *n*-Butyl fractions for each PR-10 protein.

A heating step was initially employed for rAra h 8.01, as it was found to be resistant to denaturation at 70 °C unlike the majority of *E. coli* proteins [18], but this step was abandoned when it was determined to have little effect on purity but greatly lowered yield, and was excluded from the purification protocol thereafter. All of the PR-10s remained soluble after ammonium sulfate precipitation of other proteins, bound a hydrophobic exchange resin, and were eluted with a gradient from high ammonium sulfate concentration to low-salt buffer. The robust expression and almost complete solubility of all the PR-10 proteins in this study made tremendous yields possible, as much as 120 mg of protein per liter of *E. coli* culture for rAra h 8.02. The final purification step that was employed before CD analysis, applying to the size exclusion column Superdex 200, greatly lowered the yield but was undertaken to ensure the most uniform preparations possible, as well as exchange the buffer from Tris to deionized water. A representative purification analysis, typical of all the PR-10 proteins presented in this study, is found in the SDS-PAGE of rCor a 1.02 (Figure 2A). An SDS-PAGE showing the purity of the pooled fractions from the n-butyl column for each PR-10 protein is also given (Figure 2B).

### 3.2. IgE Binding of PR-10 Proteins

**Figure 3:**
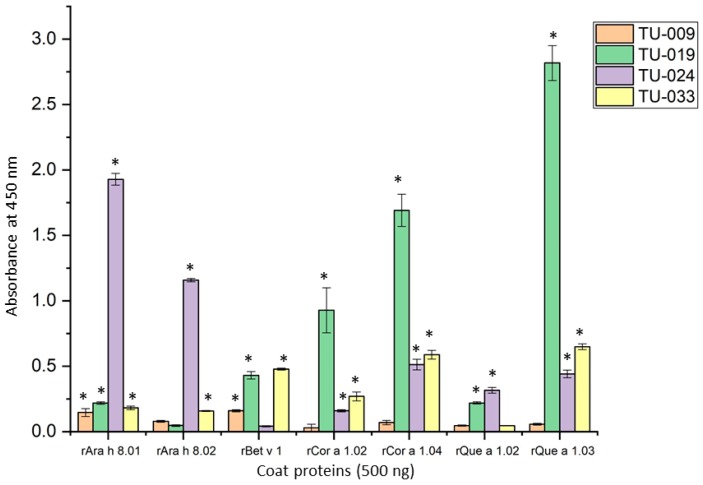
Immunoglobulin E (IgE) binding properties of the purified PR-10 proteins as determined by enzyme-linked immunosorbent assays (ELISA). The average absorbance at 450 nm after subtracting the background is plotted on the y-axis for each patient’s sera binding to 500 ng of the PR-10 protein indicated on the x-axis. The background is the average absorbance wells with the respective PR-10 protein using sera from a non-allergic individual. * Indicated all positive reactions.

We sought to characterize the IgE binding ability of our purified recombinant proteins by performing enzyme-linked immunosorbent assays (ELISAs) with sera from four patients which had been shown to bind the three PR-10 proteins in this study (rAra h 8.01, rBet v 1 and rCor a 1.04) that are also present on a commercial multiplex array, ImmunoCAP ISAC (Table 1). For the ELISAs, we set an O.D. of 0.1 above our negative controls (each PR-10 tested with sera from a non-allergic individual) as the benchmark of a positive result, and indicated all positive reactions with an * on the graph (Figure 3). All seven recombinant PR-10 proteins were able to bind the IgE of at least two patients to some extent. rAra h 8.01 was the most recognized antigen in the ELISA as it was bound by IgE in all patient samples tested, although only patient (TU-024) produced a strong signal. rCor a 1.04 was bound by IgE of all patients except TU-009 to which it had very low sIgE on the ISAC array. rBet v 1 was bound by all patients except for TU-024 but with fairly weak signals.

The other four PR-10 proteins are not present on the ISAC array so we could not confirm if these patients have IgE directed against them. Only TU-024 had a strong reaction to rAra h 8.02 and TU-019 bound rCor a 1.02 with a similar intensity. rQue a 1.02 was the most weakly bound antigen of all the PR-10s in this study, and rQue a 1.03 produced the strongest signal when bound by IgE from patient TU-019. Overall, TU-019 and TU-024 reacted most strongly to the PR-10s and, in concordance with his extremely low sIgE in the ISAC array, TU-009 had negative or very weak reactions to all the PR-10 proteins tested here.

### 3.3. Ligand Binding of PR-10 Proteins

**Figure 4:**
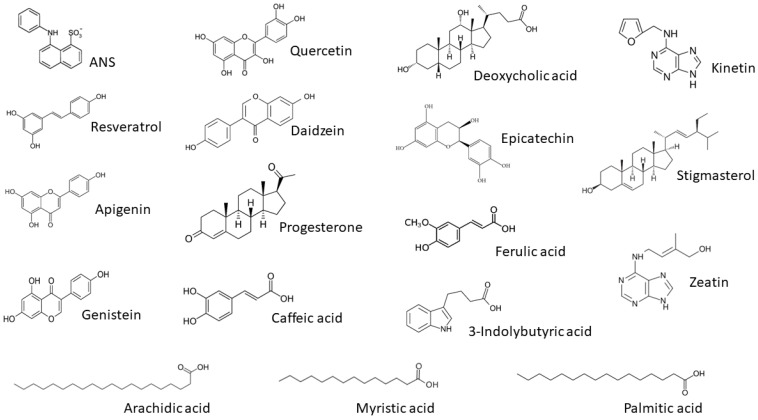
Ligands tested in fluorescence assays. Names and chemical structures of ligands found to bind PR-10 proteins in this study.

**Figure 5:**
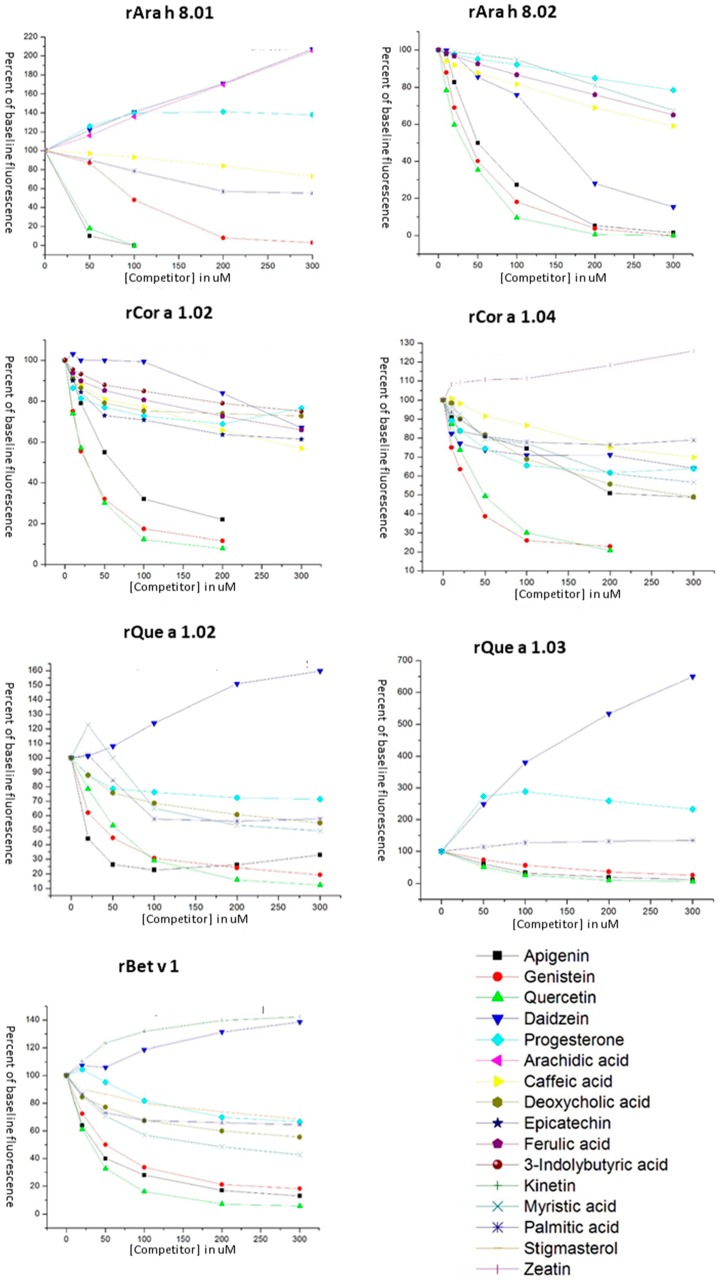
Ligand binding of PR-10 proteins. Indirect measurement of ligand binding by PR-10 proteins via the 8-anilino-1-napthalenesulfonic acid (ANS) displacement assay. Binding is measured by percent increase or decrease of the fluorescent signal of the ANS-protein complex (40 uM ANS-40 uM protein) with no competitor and recorded as a percentage of the baseline signal on the y-axis. The x-axis shows the concentration of competitor ligand in uM.

The best-characterized activity of the PR-10 protein family is binding small hydrophobic molecules such as fatty acids and flavonoids [15]. Mogensen et al. [17] first demonstrated ligand binding by Bet v 1 indirectly using the ANS displacement assay. Ligands for displacement assays (Figure 4) were selected from a variety of molecular families based on results from our previous study of rAra h 8.01, and the same concentrations of ANS and protein were used here [18]. Polyphenols, fatty acids, phenols, and plant hormones (two cytokinins, zeatin and kinetin, and one auxin, 3-indolylbutryic acid) were chosen, as well as a plant sterol (stigmasterol) and an animal sterol (progesterone) to gauge the promiscuity of the binding pocket. Of all the ligands tested, only the polyphenols (flavonoids, isoflavones, and resveratrol) and progesterone bound all of the PR-10 proteins in this study. The flavonoids apigenin, genistein, and quercetin behaved identically with all of the PR-10 proteins in this study, dramatically decreasing the fluorescence of ANS, however, the rest of the ligands had variable effects on ANS bound to the different PR-10 proteins (Figure 5). Daidzein and progesterone changed the fluorescence of ANS bound to all PR-10s tested, but some raised or lowered it, and to very different extents. For instance, 300 uM daidzein increased the fluorescence of 40 uM ANS bound to equimolar rQue a 1.03 over eight-fold, however for ANS bound to rAra h 8.01, rBet v 1 and rQue a 1.02, it only enhanced the signal by 150%–200%. Daidzein lowered the fluorescent signal of ANS that was bound to rAra h 8.02, rCor a 1.02 and rCor a 1.04. Progesterone lowered the fluorescent intensity of bound ANS for most PR-10s tested, but increased it for ANS bound to rAra h 8.01. Myristic acid had an unusual effect when introduced to ANS bound to rQue a 1.02, with a low concentration of ligand initially increasing, and then higher concentrations causing a dramatic drop in fluorescence of ANS. Overall, ANS bound to rQue a 1.03 increased in fluorescence when ligands were introduced, except for the three previously mentioned flavonoids, apigenin, genistein, and quercetin. The increase in ANS fluorescence observed is consistent with previously published studies of ligand binding by PR-10 proteins, which found that certain ligands bind at disparate sites within the hydrophobic binding pocket and do not displace ANS, but increase its signal, most likely by increasing the quantum yield [17,18]. No ligands in this study increased fluorescence of ANS bound to rAra h 8.02.

**Figure 6:**
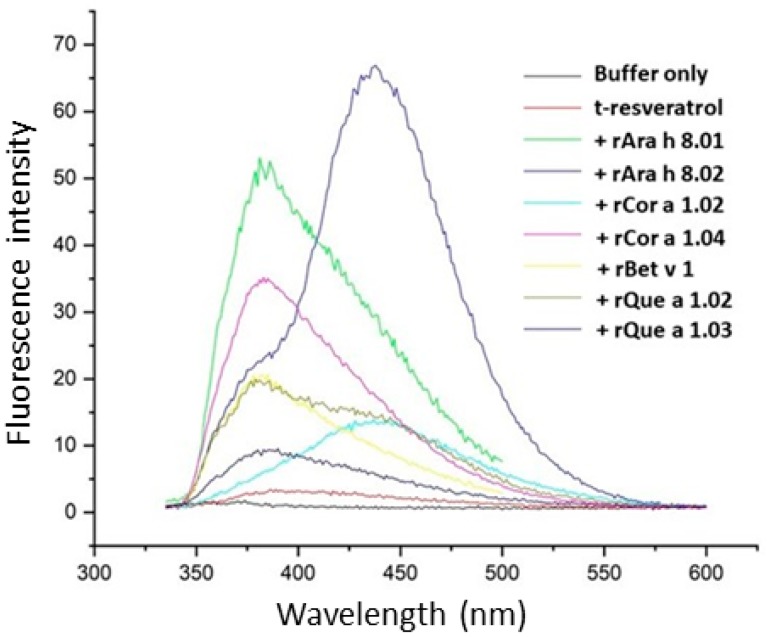
Direct measurement of t-resveratrol binding to PR-10 proteins. The fluorescence profile for each protein (20 uM) in the presence of 40 uM t-resveratrol is plotted for comparison, recorded as fluorescence intensity on the y-axis against wavelength (nm) on the x-axis.

Resveratrol itself is capable of fluorescence [32], so binding was tested directly without the addition of ANS. The emission spectra of resveratrol bound to equimolar PR-10 proteins varied in intensity, shape and profile (Figure 6). Resveratrol produced a maximal signal at 380 nm in the presence of all PR-10 proteins in this study except rAra h 8.02 and rCor a 1.02; when bound to these proteins its maxima shifted to 440 nm. Bound to rQue a 1.03, the intensity of resveratrol’s fluorescence was the weakest, about one-eighth of its signal when bound to rAra h 8.02. The difference in signal intensity of ANS bound to different PR-10 proteins was even more pronounced, with the baseline signal of ANS-rAra h 8.02 being almost 20-fold higher than of ANS-rQue a 1.03.

### 3.4. Far UV Circular Dichroism Spectroscopy

**Figure 7:**
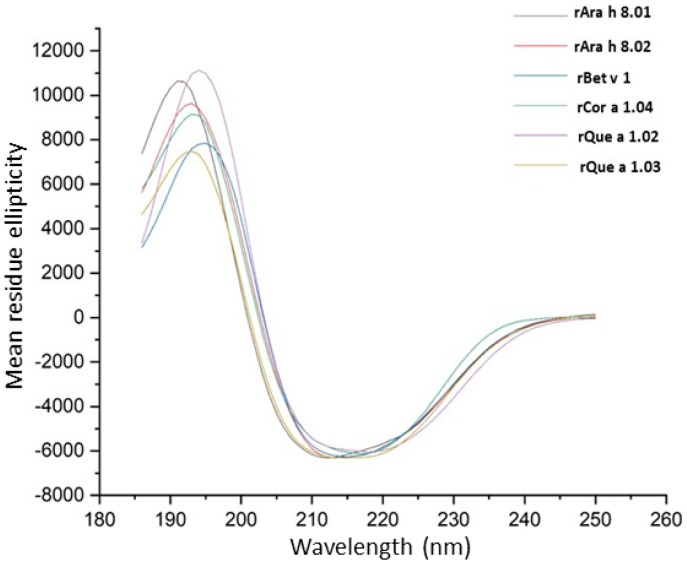
Circular dichroism (CD) spectra of PR-10 proteins. Overlay of CD spectra of PR-10 proteins reported as mean residue ellipticity on the y-axis as a function of wavelength in (nm) on the x-axis.

To date, all known structures of every studied PR-10 protein are consistent, with a seven-stranded antiparallel beta-sheet and a long C-terminal alpha-helix forming a hydrophobic cavity. Based on this universal commonality, we hypothesize that the structures of all other PR-10 proteins which are unsolved, will be the same or very similar. Based on sequence alignment with Bet v 1, the secondary structure of these PR-10s is hypothesized to be about 20% alpha helix, 40% beta sheet, and 40% random coil. The overlay of curves plotting mean residue ellipticity against wavelength (Figure 7) reveals a pattern of CD that is generally consistent with beta sheet structure and similar to the spectra obtained previously for recombinant Ara h 8 and Gly m 4 [4]; however, due to the high beta-sheet and random coil content, the usefulness of CD in determining such structures is limited [33].

## 4. Discussion

Based on our previous study of Ara h 8.01 [18] we chose to characterize other proteins in the PR-10 family of panallergens. We chose the other peanut isoform of Ara h 8 (Ara h 8.02), two representatives of hazel (Cor a 1.02 and Cor a 1.04), two representatives from white oak (Que a 1.02 and Que a 1.03), and the paradigm PR-10 protein Bet v 1 from white birch. The alignment in Figure 1 shows that there are some highly conserved residues. Mostly these are polar or charged amino acids that would not be in the hydrophobic core of the proteins. There are also nine conserved glycines which are usually in structural turns between helices or beta-sheets.

We have developed a simple three-step protocol that could serve as a general framework for the purification of recombinant PR-10 proteins from any plant, and does not rely on affinity tagging. Affinity tags, while very useful for expression of toxic or insoluble proteins and easing of purification without knowledge of a protein’s biochemistry, can interfere with downstream applications, necessitating removal by endoproteases [34]. Due to the very robust, soluble expression of these proteins, tremendous yields (50–120 mg of 90–95% homogenous protein per liter of culture) were achieved, and the basic three-step purification protocol (omitting size exclusion) can be completed in a single day. Size exclusion chromatography was not necessary for ligand and IgE binding experiments, but was performed before CD spectroscopy.

The purified recombinant PR-10 proteins all retained immunological reactivity as measured by IgE binding ELISAs. As three of the seven proteins appear on the ISAC commercial multiplex array, we were able to compare directly the results of the two experiments for the four patients we tested. A side-by-side comparison of the data for rAra h 8.01 shows consistency between the two methods. TU-024 had moderate sIgE to rAra h 8.01 on the ISAC array and a strong signal in the ELISA, while the other three patients had low sIgE on the ISAC and weak signals in the ELISA. The results for rCor a 1.04 and rBet v 1 were somewhat ambiguous. The signals in ELISA against rCor a 1.04 for patients TU-019 and TU-024 were about equal, but TU-024 only had about one-fifth the titer as TU-019. TU-019 had extremely high sIgE to rBet v 1 as measured by ISAC (84.7 kU_A_/L) but a fairly weak reaction in the ELISA, and TU-024 had moderate levels of sIgE in the ISAC array (7.5 kU_A_/L) and a negative result in the ELISA. Recombinant proteins are often less immunoreactive than natural proteins due to lack of post-translational modifications or improper folding [35], however Bet v 1 is present in recombinant form on the ISAC chip as well, and both are the same immunodominant isoform, rBet v 1.01A [36]. Moreover, recombinant Bet v 1 has long been known to be highly reactive in both diagnostic immunoassays and immunotherapy (reviewed in [37]). As we are confident that our purified proteins retained their proper secondary structure as shown by CD, we do not believe that the lower affinity for rBet v 1 in the ELISA is due to improperly folded proteins.

The flavonoids (quercetin, genistein, and apigenin) behaved identically in the ligand-binding assay with all seven of the recombinant PR-10 proteins. The addition of flavonoid to the PR-10-ANS complex caused a sharp decrease in fluorescence, with the signal either completely extinguished or with an intensity less than 20% of the baseline signal. This result was consistent for all three ligands competing with ANS for binding to all seven PR-10 proteins, with the exception of apigenin as a competitor for rCor a 1.04, which only reduced the ANS signal by half. Daidzein, another flavonoid with an almost identical structure, did not behave like the other three flavonoids but did affect the signal of ANS when bound to all seven proteins. Daidzein was generally a weaker competitor for ANS than the other three flavonoids, yet it strongly enhanced the signal when added to the rQue a 1.03-ANS complex, indicating it likely binds to a different site within that protein’s pocket than the sites it binds in the other proteins.

The strength of binding by the flavonoids suggests that these molecules may be the true biological ligands of PR-10 proteins, and that weakly binding molecules like fatty acids may be identified in this screen due to the relative promiscuity of the PR-10 hydrophobic pocket. The flavonoids are 15-carbon polyphenolics that are usually found as glycosides. They are ubiquitous in the plant kingdom, and are important pigments and regulators, with their mechanism of action in the plant pathogen defense response possibly being enzyme inhibition [25]. PR-10 proteins may act as transporters or reservoirs for the flavonoids, and facilitate their accumulation when a plant is stressed or infected.

We present this method as a flexible generalized protocol that is amenable to recombinant PR-10 proteins from pollen, legume, and nut sources. We believe it should be applicable to PR-10 proteins from fruits and vegetables as well. Recombinant proteins have some limitations in immunological research as they lack higher ordered structures and have post-translational modifications to amino acids that are difficult or impossible to produce in an *E. coli* cell [38], but their use is necessary as separation of the various isoforms of PR-10 proteins within a plant would be nearly impossible. Also, PR-10 proteins are often present in very low abundance within the plant extract [39]. The ability to rapidly produce recombinant PR-10 proteins from a variety of sources will facilitate research into the immunology, structures, and biological functions of this important class of allergens and could be a readily modifiable agent for immunotherapy.

## Figures and Tables

**Table 1:** Patient information.

Patient ID	Age	Sex	Ara h 8.01	Bet v 1	Cor a 1.04	Known Food Allergies
TU-009	56	M	1.04	2.76	0.63	Walnut, pecan, almond, brazil nut, pine nut, hazelnut, macadamia nut, apricot, apple, pear, plum, similar fruits
TU-019	36	F	1.61	84.7	35.0	Soy, peach, pear, apple, cherry, pine nut
TU-024	24	F	8.04	7.5	7.47	Cashew, peanut, walnut, pecan, other tree nuts
TU-033	26	F	1.28	14.7	5.14	Peanut, brazil nut, other tree nuts, mango, peach, plum, melons

**Table 2:** Recombinant PR-10 protein information. Isoelectric points (pI) and molecular weights (MW).

Protein Name	Calculated MW (Da)	Calculated PI	[Ammonium Sulfate]
rAra h 8.01	16,952	4.955	60% of saturation
rAra h 8.02	16,412	5.003	50% of saturation
rBet v 1.01	17,571	5.411	60% of saturation
rCor a 1.02	17,417	5.931	60% of saturation
rCor a 1.04	17,581	6.448	50% of saturation
rQue a 1.02	17,303	5.741	45% of saturation
rQue a 1.03	17,478	5.402	40% of saturation

## Supplementary Materials

The following are available online at https://www.mdpi.com/2304-8158/8/12/609/s1, Figure S1: Tandem Mass Spectrometry Data of PR-10 proteins.
