# Supplementary material for: Purification and Characterization of Pathogenesis Related Class 10 Panallergens
Source: Foods. 2019 Nov 22;8(12):609. doi: 10.3390/foods8120609 (PMC6963334; doi:10.3390/foods8120609)
Supplement: Supplementary file 1 [file foods-08-00609-s001.pdf]

Supplemental Figure S1: Tandem Mass Spectrometry Data of PR-10 proteins

	Subgroup (#)	Spectra (#)	Distinct Peptides (#)	Distinct Summed MS/MS Search Score	% AA Coverage	Total Protein Spectral Intensity	Database Accession #	Protein Name
1	1.1	64	14	259.11	76.8	5.69e+009	7	Que a 1.03
1	1.2	52	14	256.21	89.3	3.75e+009	6	Que a 1.02
2	2.1	52	13	227.91	75	2.09e+009	4	Cor a 1.02
3	3.1	83	12	211.98	74.5	1.57e+009	2	Ara h 8.02
4	4.1	24	11	201.55	73.9	2.48e+009	5	Cor a 1.04
5	5.1	57	11	189.50	61.2	6.82e+009	3	Bet v 1
6	6.1	38	12	189.15	82.1	1.97e+009	1	Ara h 8.01
**Totals:**	370	87						

Ara h 8.01

```
1  MGVFTFEDEI TSTVPPAKLY NAMKDADSIT PKIIDDVKSV EIVEGNGGPG TIKKLTIVED GETKFILHKV ESIDEANYAY 80
81 NYSVVGGVAL PPTAEKITFE TKLVEGPNGG SIGKLTLKYH TKGDAKPDEE ELKKGKAKGE GLFRAIEGYV LANPTQY    157
```

The matched peptides cover **82%** (130/157 AA's) of the protein.
Protein Name: **Ara h 8.01**
NCBInr.Jane_1 Accession #: **1**
MS Digest Index #: **1**
Protein MW: **16951.9** Da
Amino Acid Composition: **A11 D9 E16 F5 G17 H2 I12 K18 L10 M2 N6 P9 Q1 R1 S6 T13 V12 Y7**

Ara h 8.02

```
1  MGVHTFEEES TSPVPPAKLF KATVVDGDEL TPKLIPAIQS IEIVEGNGGP GTVKKVTAVE DGKTSYVLHK IDAIDEATYT 80
81 YDYTISGGTG FQEILEKVSF KTKLEAADGG SKIKVSVTFH TKGDAPLPDE VHQDVKQKSQ GIFKAIEGYV LSN        153
```

The matched peptides cover **75%** (115/153 AA's) of the protein.
Protein Name: **Ara h 8.02**
NCBInr.Jane_1 Accession #: **2**
MS Digest Index #: **2**
Protein MW: **16412.2** Da
Amino Acid Composition: **A10 D10 E13 F6 G15 H4 I11 K16 L8 M1 N2 P8 Q5 S10 T14 V15 Y5**

Bet v 1.01

```
1  MGVFNYETET TSVIPAARLF KAFILDGDNL FPKVAPQAIS SVENIEGNGG PGTIKKISFP EGFPFKYVKD RVDEVDHTNF 80
81 KYNYSVIEGG PIGDTLEKIS NEIKIVATPD GGSILKISNK YHTKGDHEVK AEQVKASKEM GETLLRAVES YLLAHSDAYN 160
```

The matched peptides cover **61%** (98/160 AA's) of the protein.
Protein Name: **Bet v 1**
NCBInr.Jane_1 Accession #: **3**
MS Digest Index #: **3**
Protein MW: **17570.5** Da
Amino Acid Composition: **A11 D9 E14 F8 G14 H4 I13 K15 L9 M2 N9 P8 Q2 R3 S11 T9 V12 Y7**

Supplemental Figure S1: Tandem Mass Spectrometry Data of PR-10 proteins

Cor a 1.02

1 MGVFNYETES TSVIPAARLF KAFILDGNNL IPKVAPQAVS SVENVEGNGG PGTIKKITFS EGSPFKYVKE RVEEVDHTNF 80
81 KYSYTVIEGG PVGDKVEKIC NEIKIVAAPD GGSILKISNK YHTKGDHEVD AEHIKGGKEK VEGLFRAVEA YLLAHSDAYN 160

The matched peptides cover **75%** (121/160 AA's) of the protein.
Protein Name: **Cora 1.02**
NCBInr.Jane_1 Accession #: **4**
MS Digest Index #: **4**
Protein MW: **17416.3** Da
Amino Acid Composition: **A12 C1 D7 E16 F7 G16 H5 I12 K16 L7 M1 N9 P7 Q1 R3 S10 T7 V16 Y7**

Cor a 1.04

1 MGVFCYEDEA TSVIPPARLF KSFVLDADNL IPKVAPQHFT SAENLEGNGG PGTIKKITFA EGNEFKYMKH KVEEIDHANF 80
81 KYCYSIIEGG PLGHTLEKIS YEIKMAAAPH GGGSILKITS KYHTKGNASI NEEEIKAGKE KAAGLFKAVE AYLLAHPDAY 160
161 C 161

The matched peptides cover **74%** (120/161 AA's) of the protein.
Protein Name: **Cora 1.04**
NCBInr.Jane_1 Accession #: **5**
MS Digest Index #: **5**
Protein MW: **17580.7** Da
Amino Acid Composition: **A18 C3 D5 E16 F8 G15 H7 I13 K17 L10 M3 N7 P8 Q1 R1 S8 T7 V6 Y8**

Que a 1.02

1 MGVFTHESQE TSVIAPARLF KALFLDSDNL IQKVLPQAIK STEIIEGNGG PGTIKKITFG EASKFKYAKH RIDALDPENC 80
81 TYSFSVIEGD ALTVMESVST EIKCVASPDG GSIMKSTTKY QTKGDFQLKE EQIQAAIEKA AGLLKAVEAY LVAHPDLYK 159

The matched peptides cover **89%** (143/159 AA's) of the protein.
Protein Name: **Que a 1.02**
NCBInr.Jane_1 Accession #: **6**
MS Digest Index #: **6**
Protein MW: **17302.5** Da
Amino Acid Composition: **A16 C2 D8 E13 F7 G11 H3 I13 K16 L12 M3 N3 P6 Q7 R2 S12 T11 V9 Y5**

Que a 1.03

1 MGVFTYESED ASVIPPARLF KAFVLDSDNL IPKVVPQALK STEIIEGNGG PGTIKKITFG EGSHLKHAKH RIDVIDPENF 80
81 TYSFSVIEGD ALFDKLENVS TETKIVASPD GGSIVKSTSK YQTKGDFQLT DELIRGGKEK ASEVFKAVEA YLVAHPDLYK 160

The matched peptides cover **76%** (123/160 AA's) of the protein.
Protein Name: **Que a 1.03**
NCBInr.Jane_1 Accession #: **7**
MS Digest Index #: **7**
Protein MW: **17477.5** Da
Amino Acid Composition: **A11 D11 E13 F9 G13 H4 I12 K16 L11 M1 N4 P8 Q3 R3 S13 T10 V13 Y5**
